# “*Academic mountaineering*” in medicine: competition vs. cooperation and medical professionalism

**DOI:** 10.3389/fmed.2025.1682480

**Published:** 2025-10-16

**Authors:** Patrícia Teixeira Costa, Paulo Cesar Massucatto Colbachini, Lucas Silva Mello, Andrea de Melo Alexandre Fraga, Mônica Cássia Firmida, Fernando Augusto Lima Marson

**Affiliations:** ^1^Laboratory of Genetics and Molecular Biology, São Francisco University (USF of the Portuguese Universidade São Francisco), Bragança Paulista, São Paulo, Brazil; ^2^Laboratory of Clinical Microbiology and Genetics, São Francisco University (USF of the Portuguese Universidade São Francisco), Bragança Paulista, São Paulo, Brazil; ^3^LunGuardian Research Group — Epidemiology of Respiratory and Infectious Diseases, São Francisco University (USF of the Portuguese Universidade São Francisco), Bragança Paulista, São Paulo, Brazil; ^4^Department of Pediatrics, School of Medicine, Pontifical Catholic University of Campinas (PUC-Campinas of the Portuguese Pontifícia Universidade Católica de Campinas), Campinas, São Paulo, Brazil; ^5^Department of Pediatrics, School of Medical Sciences, University of Campinas (Unicamp of the Portuguese Universade de Campinas), São Paulo, Brazil; ^6^Department of Integrated Medical Sciences, University of the State of Rio de Janeiro (UERJ of the Portuguese Universidade do Estado do Rio de Janeiro), Cabo Frio, Rio de Janeiro, Brazil

**Keywords:** *academic mountaineering*, ethics, medical training, mental health, professionalism

## 1 Introduction

**Figure 1:**
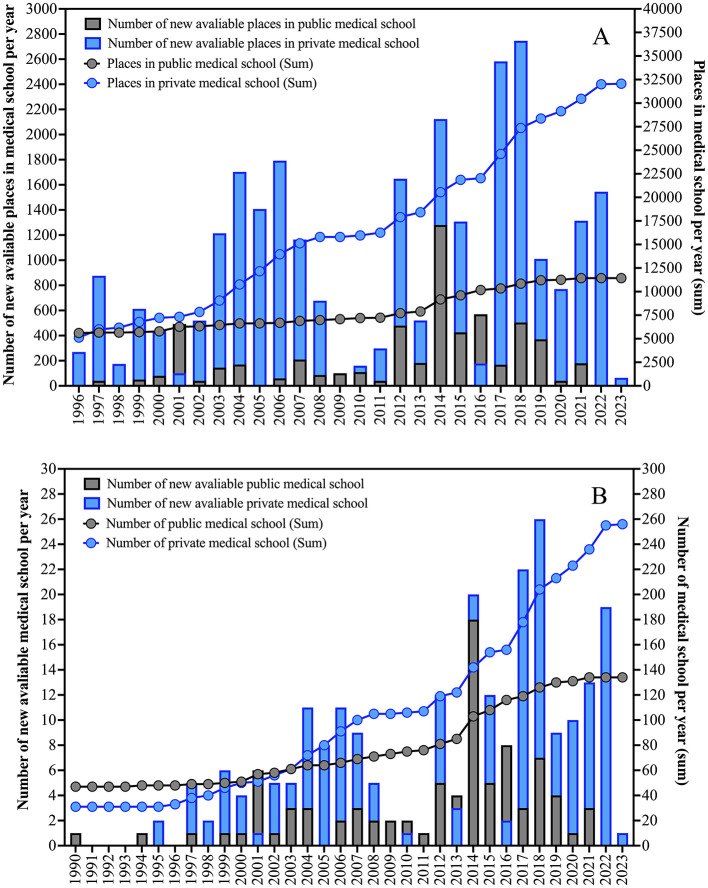
**(A)** Distribution of the number of new places in medical schools in Brazil between 1996 and 2023. **(B)** Distribution of the number of new medical schools in Brazil between 1990 and 2023. The data is presented in absolute numbers according to the profile of the medical school (public and private). The data presented was collected from the Federal Council of Medicine of Brazil from the platform Radiography of Medical Schools with data updated on April 22, 2024.

Medical training in Brazil faces significant challenges due to the rapid expansion of medical schools and increasing competition. Currently, there are 390 medical schools, predominantly private and concentrated in the Southeast region (1) (Figure 1). From 1990 to 2024, the number of professionals more than quadrupled, increasing from 131,278 to 598,573. However, the geographical distribution remains uneven, and the number of residency positions has not kept pace.

In this competitive environment, many students focus on accumulating credentials rather than engaging in meaningful learning. The National Medical Residency Exam strongly influences their choices, driving them to engage in various activities to enhance their *résumés* (2). The phenomenon we term “*academic mountaineering*” has not been previously defined in the literature. We propose it as a novel conceptual contribution: a pattern of behavior in which students engage in strategic accumulation of academic, research, and extracurricular achievements primarily to satisfy evaluation criteria, rather than as an expression of intrinsic interest or ethical commitment. This promotes a superficial, individualistic approach to training, undermining professionalism and the holistic development of future physicians. Theoretical support for this framework can be drawn from sociological models of institutional rationality, where individuals adjust their actions to maximize perceived rewards under systemic constraints (3).

This paper examines how “*academic mountaineering*” affects cognitive learning, ethics, and mental health. It explores factors such as competition, self-promotion, and the hidden curriculum, while also addressing ethical concerns. Lastly, we propose strategies to counter these issues and promote a more balanced, ethics-driven medical education.

## 2 Medical professionalism and “academic mountaineering”: opposing forces in undergraduate education

Although there is no universal definition of medical professionalism (4), it extends beyond technical expertise. It encompasses behaviors and attitudes rooted in ethics, integrity, altruism, and social responsibility, promoting good practices in comprehensive healthcare and lifelong learning. A key reference on the subject, the guide “*Medical Professionalism in the New Millennium: A Physician Charter*,” emphasizes three fundamental principles: patient wellbeing, patient autonomy, and social justice. It also highlights competencies such as quality of care, trust, professional responsibility, and ethics (5). Ang defined medical professionalism as a set of behaviors, attitudes, and practices covering doctor-patient interactions, colleague relationships, health maintenance, integrity, financial and business practices, and high-quality clinical care (6). Other definitions align with these values, emphasizing the skills and competencies that support professional best practices.

**Figure 2:**
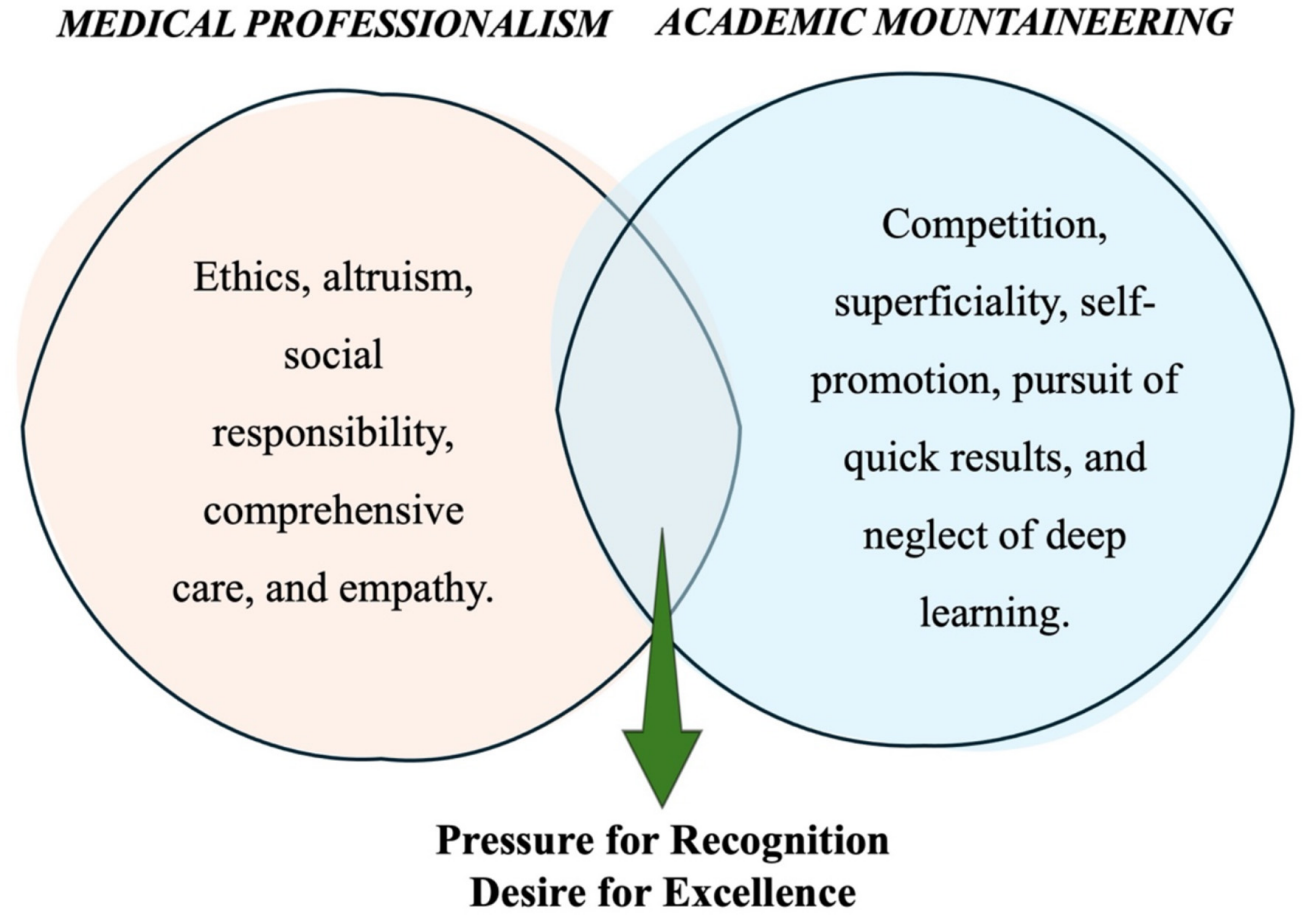
Professional profile model associated with medical professionalism and “*academic mountaineering*” through the pressure for recognition and the desire for excellence.

In contrast to professionalism, “*academic mountaineering*” undermines both technical-scientific and humanistic training, hinders collaboration, and compromises commitment to patients and society. It also increases the risk of unethical behavior and negatively affects students' mental health. While lapses in professionalism can occur during learning, educators play a key role in addressing these issues through ethical discussions, real-world problem-solving, and mentorship (7, 8). To counteract unprofessional behavior, medical training should emphasize interdisciplinary learning, ethical reflection, and active student engagement. The hidden curriculum—the set of unwritten norms and values transmitted through institutional culture—plays a crucial role in perpetuating the values associated with “*academic mountaineering*” and must be explicitly addressed (9). The professional profile model associated with medical professionalism and “*academic mountaineering*” is presented in Figure 2.

## 3 “*Academic mountaineering*” and its impact on student mental health

Mental health issues, reduced quality of life, and diminished empathy in medical students—primarily due to academic overload—have been widely studied in recent years. These studies aim to diagnose the situation and identify preventive and control measures (10, 11).

A Brazilian meta-analysis reviewed 59 qualitative (mostly observational and cross-sectional), involving 18015 medical students. It found high rates of mental disorders, including depression [30.6%], common mental disorders [31.5%], burnout [13.1%], problematic alcohol use [32.9%], stress [49.9%], poor sleep quality [51.5%], excessive daytime sleepiness [46.1%], and anxiety [32.9%]. These issues are linked to a mix of individual, academic, and institutional factors, particularly excessive teaching hours, academic overload, and competitiveness, which lead to sleep deprivation and reduced leisure time (12).

This constant competition and fear of being “left behind” create stress, transforming the academic journey into a series of struggles rather than fostering excitement and self-confidence. Social networks amplify this by showcasing success stories, often with exaggerated narratives, which heighten insecurity. Stress from performance pressure and peer comparison is linked to common health issues and can lead to depression, anxiety, and burnout (13).

Addressing this issue requires a systemic response, including structured psychological support, academic mentoring, and institutional policies that promote student wellbeing. Medical schools should implement wellness initiatives that encourage balance, self-care, and realistic goal-setting, reducing the pressure associated with excessive competition.

## 4 Scientific production: target or path?

Scientific inquiry is a fundamental component of medical education, fostering critical thinking and problem-solving skills. Research encourages evidence-based practice and lifelong learning. However, “*academic mountaineering*” often distorts its purpose, turning it into a tool for *résumé* enhancement rather than a meaningful investigation of scientific questions, leading to superficial contributions to medical knowledge.

Participating in research during undergraduate studies helps students develop skills such as asking questions, searching for academic information, critically reading articles, developing critical and argumentative thinking, writing scientific papers, and producing knowledge. These skills are valuable for everyday medical practice, even for those not focused on a research career.

Initiatives that promote scientific production during undergraduate studies help shape well-rounded professionals with investigative skills and a critical perspective, essential for evidence-based practice and innovation (14). Developing skills in scientific methodology, data analysis, and academic communication opens opportunities for those pursuing basic or clinical research. Research experience can also spark interest in specific fields and guide specialization choices. Publications and presentations at scientific events enhance communication skills and strengthen a curriculum vitae for selection in lato sensu or stricto sensu postgraduate courses.

Both research and teaching require dedication and time to develop critical and connective thinking, contrasting with the short-term focus prevalent in a society driven by quick solutions and immediate results. However, students' motivations for engaging in research are multifactorial. According to Merton's sociological framework, scientific engagement often emerges from a complex interplay between intrinsic motivations (curiosity, commitment to knowledge) and institutional incentives (career advancement) (15). Contemporary medical training faces the challenges of managing the rapid growth of scientific knowledge while resisting the superficiality of the “infocracy,” where information overload and pressure for productivity undermine in-depth learning and empathy-based practice, as philosopher Byung-Chul Han warns (16).

The benefits of teaching, extension, and research require gradual learning, time, and genuine dedication, characterized by commitment, determination, and perseverance. This contrasts with “*academic mountaineering*,” which seeks quick results and focuses solely on production.

## 5 Concrete manifestations of “*academic mountaineering*”: representative cases

The phenomenon of “*academic mountaineering*” becomes evident in a wide range of student behaviors that, while often socially rewarded, reveal a fundamentally instrumental approach to academic activities (Table 1). These examples are not isolated practices but manifestations of deeper structural tensions within medical education: competition vs. cooperation, quantity vs. quality, and personal image vs. genuine learning. One of its most recognizable manifestations lies in the pursuit of research projects driven not by curiosity or commitment to scientific inquiry, but with the sole purpose of multiplying publications and conference abstracts. For example, a student may join three simultaneous research groups, with little real involvement in any of them, just to have his or her name appear in multiple abstracts. Resorting to predatory journals or fragmenting research findings into multiple and sometimes redundant articles are other expressions of this tension, where the symbolic value of “having published” outweighs the actual contribution to science.

**Table 1:** Representative cases of “*academic mountaineering*” in medical education: behaviors, instrumental motivations, and negative impacts (41–53).

**Category**	**Behaviors**	**Negative impact**
Participation in research projects for instrumental purposes	Engagement in multiples projects without genuine interest, in order only to accumulate publications; multiple paper presentations without real understanding of study goals and methodology.	Superficial scientific production; loss of the formative value of academic research.
“Curriculum-driven” engagement in complementary activities	Participate in extracurricular activities, such as academic leagues, extension projects and others, with minimal involvement, only for curriculum improvement.	Shallow commitment; scattered attention; diminished educational and supportive value of such activities.
Inflated academic production	Publication in predatory journals; slicing data to produce multiple papers; data manipulation.	Superficial and/or redundant scientific output; compromised research quality; unethical practices.
Strategic volunteering	Participation in campaigns and volunteer activities primarily to enhance the curriculum.	Distorted motivations behind social engagement; reduced credibility of volunteer work.
Dysfunctional competitiveness	Avoiding collaboration; withholding information from peers (e.g., materials, data, results, and academic opportunities).	Promotion of individualistic culture; hindered learning due to lack of cooperation.
Excessive pursuit of courses and certificates	Enrolling in numerous online courses, workshops, and events to collect certificates without meaningful learning.	Quantity-over-quality mindset; shallow understanding of topics.
Utilitarian networking	Approaching peers and/or superiors solely to obtain project opportunities, recommendation letters, positions, and others.	Culture of utilitarianism; superficial relationships; erosion of the mentoring and tutoring experience.
Focus on external metrics	Defining success by the number of publications, certificates, event participations, and similar indicators.	Increased anxiety and burnout; diminished education value of these activities.
Academic ego	Disproportionate pride in achievements; excessive sharing on social media.	Heightened anxiety and burnout; prioritization of personal image over genuine learning.

Another frequent manifestation is superficial engagement in extracurricular activities. Students may enroll in academic leagues or extension projects only to add them to their curriculum vitae, without sustained participation or genuine commitment. In some cases, a student may show up at only one or two activities of a year-long program but still list it as part of his or her academic record. Similarly, competitive behaviors such as hiding materials from colleagues or refusing collaboration reflect how “*academic mountaineering*” erodes a culture of shared learning. For instance, a student preparing for a residency exam might withhold helpful resources from peers to preserve a perceived advantage.

The excessive pursuit of certificates is also emblematic. It is not unusual for students to attend dozens of online courses and workshops within a short period, rapidly accumulating certificates but retaining little meaningful knowledge. A similar logic applies to opportunistic networking: approaching senior faculty only to secure letters of recommendation or project opportunities, rather than for mentorship or learning. These practices distort the formative dimension of academic relationships, reducing them to transactional exchanges.

Finally, the focus on external markers of success—publications, certificates, event participation, or positions held—becomes a symbol of prestige that feeds personal image rather than professional development. Social media often amplifies this effect: some students may share every minor accomplishment online, reinforcing a culture of comparison and ego-driven recognition. This creates a vicious cycle, fueling competition and social comparison among peers, while deepening superficial engagement and individualistic attitudes.

In sum, these representative cases show how “*academic mountaineering*” is not just about individual choices but about systemic pressures and incentives that privilege visibility and accumulation over substance. Recognizing these tensions is essential for building strategies that restore balance between formative learning, collaboration, and authentic professional growth.

## 6 “Scientific ego”: risks to science and ethics

The pressure for recognition and publication contributes to what may be termed a “scientific ego”—a disposition where personal visibility takes precedence over the collaborative nature of science (17). “*Academic mountaineering*” fosters a culture of self-promotion, where individual recognition is prioritized over collaborative learning. This behavior is reinforced by faculty expectations and institutional reward structures that emphasize publications and accolades over professional integrity. As a result, the true purpose of scientific training—cooperative knowledge-building for the advancement of society and medical practice—is lost. Instead of collaboration, conflicts arise, and the process becomes a race for recognition, often at the expense of academic integrity. This environment increases the risk of unethical behavior, such as data manipulation and the omission of flaws. Empirical studies have shown that environments of high publication pressure are associated with increased incidence of questionable research practices (18).

One way to more accurately assess an author's impact is not through reliance on a single evaluation method, which may provide useful feedback but also has inherent limitations, but rather through the combination of multiple complementary approaches. These may include the H-index and i10 index, Field-Weighted Citation Impact and Category Normalized Citation Impact, Highly Cited Papers, Citation Percentile, Author-level Eigenfactor Score, Author Impact Factor and Journal Impact Factor, N-Index, Altmetric Donut and Altmetric Attention Score, Co-authorship Network Analysis, Awards and Honors, and the Author Contribution Index (19).

Medical schools must promote academic integrity by fostering collaboration, implementing transparent authorship guidelines, providing mentorship in research ethics, and valuing meaningful academic work over sheer output.

## 7 Self-promotion and the risk of superficial learning

The desire for recognition aligns with the widespread habit of self-promotion, particularly amplified by social networks. These platforms enable students and professionals to easily share their achievements, which, in itself, is not inherently negative. However, the issue arises when this sharing becomes exaggerated, distorted, or decontextualized. Many students treat research publications as “prestige markers” rather than opportunities for meaningful growth. This behavior can be understood within the framework of impression management theory, where individuals actively construct a favorable image of themselves to gain social or institutional advantage (20).

Federal Council of Medicine Resolution No. 2336/2023 regulates medical advertising in Brazil (21). This new resolution updates Federal Council of Medicine Resolution No. 1974/2011. It allows doctors to publicize their work on social networks, advertise the equipment available in their practices, and, for educational purposes, use images of their patients or images from a photo bank. As rapporteur Emmanuel Fortes described—“*Previously, we had mostly prohibitions. Now, we embrace the freedom to advertise, but responsibly and without sensationalism*.” Although this legal shift grants more freedom, it also increases the responsibility of both medical professionals and students to maintain ethical standards in self-presentation.

Educational institutions should prioritize authentic engagement over symbolic participation. Ethical awareness campaigns and faculty mentorship can guide students to value deep learning and professional integrity rather than focusing solely on external recognition.

## 8 The use of artificial intelligence (AI) in medical practice

AI is becoming more integrated into medical education, offering valuable tools for research, clinical decision-making, and knowledge synthesis. However, its misuse poses risks to academic integrity and critical thinking. Relying on AI-generated essays, research summaries, and diagnostic tools without fully understanding the underlying concepts can hinder learning and raise ethical concerns about authorship, plagiarism, and misinformation.

The unrestricted use of powerful chatbots (e.g., ChatGPT, Google Gemini, Microsoft Copilot, Perplexity, and Claude) can lead to serious ethical issues, such as the creation of elaborate fake news, similar to what was seen in the global anti-vaccine movement (22–24). This can foster doubt about science and scientific progress, as well as generate content that may be interpreted more intensely than intended (25). Also, when used without critical engagement, AI-generated content can contribute to plagiarism, misinformation, and loss of authorship integrity (26).

In the healthcare sector, not all professionals are sufficiently trained in using AI technologies, which can lead to informational or technical errors. For doctors, improper use of AI can undermine their relationship with patients, potentially causing fear and confusion instead of fostering trust and understanding (25).

For medical students, the emphasis on quantity over quality often encourages the misuse of AI technologies, with the goal of quickly building a strong foundation for the National Medical Residency Examination. This leads to the creation of superficial summaries, basic responses to essay questions, and even entire academic articles. While AI technologies can generate convincing scientific articles that resemble human-written work, errors often appear in referencing and semantics, which can be detected by experienced readers (27, 28). These articles not only lack genuine student knowledge but may also be accepted for publication, potentially cited by other researchers, or presented at scientific congresses, where they could mislead both professionals and the public.

In medical schools, the lack of formal training in digital literacy and AI ethics exacerbates the problem. Medical schools should establish guidelines for the responsible use of AI, encouraging students to critically engage with AI-generated content rather than passively accepting it. Training in digital literacy and academic integrity is essential to uphold high ethical standards in medical education.

Students may use these tools to produce seemingly sophisticated work for residency applications, contributing to the illusion of competence without genuine understanding. This technological shortcut aligns with the logic of “*academic mountaineering*,” where the final product is valued over the process of learning. To mitigate these risks, institutions must develop clear guidelines for ethical AI use, integrate digital health and AI literacy into curricula, and foster critical reflection on technology's role in professional development.

## 9 Implications for medical practice

Physicians trained in systems that prioritize credential accumulation over technical and humanistic learning may be ill-prepared for clinical practice. Superficial medical training can have serious consequences for patient care. Strong doctor-patient relationships require empathy, effective communication, and critical thinking—qualities often neglected by “*academic mountaineering*.” Studies in medical education have shown that empathy tends to decline throughout medical school, particularly in competitive and high-stress environments, affecting not only patient satisfaction but also diagnostic accuracy and treatment adherence (29).

Moreover, the lack of proper training hampers comprehensive healthcare, compromising professionals' ability to make accurate diagnoses, provide optimal treatments, and manage complex clinical situations. This often results in mechanized, depersonalized care, which can jeopardize patients' health and even their lives. The emphasis on procedural knowledge over reflective practice also limits the physician's adaptability in diverse clinical settings, including underserved or resource-limited environments (30).

To ensure high-quality healthcare, medical education must emphasize patient-centered training, ethical reflection, and professional mentorship. Moving away from competitive frameworks will better prepare doctors for the realities of clinical practice.

## 10 Contextualizing “*academic mountaineering*” from other perspectives

The concept of “*academic mountaineering*” practices is not limited to Brazil. In *Arthroscopy: The Journal of Arthroscopic and Related Surgery*, the editorial “*Publish or Perish Promotes Medical Literature Quantity Over Quality”* highlights the problem of the “publish or perish” phenomenon in academia. This phenomenom is characterized by the rapid and continuous production of academic work, often prioritizing quantity over quality, as a means to sustain or advance an academic career. Publish or perish is reinforced by several incentives, including those linked to the Accreditation Council for Graduate Medical Education, such as program admission, faculty promotion, accreditation, and professional recognition. While these mechanisms may ocassionally promote quality, their primary effect is to encourage quantity. It has therefore been proposed that the training of medical scientists and clinicians should emphatize the transmission of expertise in research methods and the evaluation of scholarly authorship through validated quality-based metrics (31).

The publish or perish phenomenon can also lead to the creation of fraudulent or predatory scientific and medical journals, due to the rise in the number of articles, as well as an increase in the number of authors listed, which can ultimately lead to authorship issues. Additionally, this is not restricted only to medical schools, given that other subjects or areas within the health sector may be compromised, such as in biomedical sciences (19).

## 11 International comparative dimension: how other countries have addressed “*academic mountaineering*”

The discussion about prioritizing quantity over quality in scientific and educational outputs is not unique to Brazil—several jurisdictions have implemented policy and cultural changes aimed at reducing incentives for merely quantitative production and revaluing teaching, integrity, and student wellbeing. One widely disseminated international strategy is the reform of scientific output evaluation criteria: the San Francisco Declaration on Research Assessment recommends avoiding the use of the *journal impact factor* as a proxy for individual merit and encourages more qualitative and contextual approaches in career evaluation and academic promotion. Adoption of Declaration on Research Assessment principles by universities and funding bodies has been used as a tool to discourage practices that fuel the “publish or perish” culture (32, 33).

Another change that had an indirect impact on “*résumé-building*” behaviors was the modification of student assessment systems. In the United States of America, the transition of the United States Medical Licensing Examination Step 1 to a *pass/fail* format sought to reduce reliance on a single numerical metric that drove excessive competition among students and a greater emphasis on extracurricular activities to compete for residency positions. Recent studies have examined the effects of this change on residency selection, student perceptions, and the potential shifting of pressure toward other metrics, showing that assessment reforms require complementary policies to prevent unintended displacements of pressure (34–36).

In addition, *promotion and tenure* programs have been revised in countries such as the United States of America and the Netherlands to incorporate broader criteria—recognition of teaching, outreach, leadership, and social impact—rather than focusing exclusively on number of publications or journal impact factors. Reviews and institutional guidelines on promotion practices indicate that changes in evaluation metrics must be accompanied by incentives and recognition structures that make academic careers more diverse and ethical (37).

Another intervention with evidence on student wellbeing is the adoption of *pass/fail* grading systems throughout medical school, which, in systematic reviews, has been associated with improved wellbeing without consistent harm to academic performance; however, effects on curricular behaviors (such as greater or lesser propensity toward “activity accumulation”) vary according to local context and selection mechanisms. Thus, isolated changes tend to shift pressures rather than resolve them, unless they are part of an integrated policy package (revision of selection criteria, mentorship, mental health support, and formal recognition of teaching and community activities) (38, 39).

Finally, it is recommended that Brazilian national and institutional policies consider these international lessons: (i) adopt responsible evaluation principles (e.g., Declaration on Research Assessment) for assessing faculty and researchers; (ii) revise criteria for residency selection and career progression to value teaching, community service, and integrity; (iii) implement structured wellbeing and mentorship programs; and (iv) monitor unintended consequences (pressure shifting). International experience shows that isolated changes (e.g., making an exam *pass/fail*) can reduce one source of pressure but require complementary measures to prevent competition and “*academic mountaineering*” from migrating to other metrics (32–39).

## 12 Facing “*academic mountaineering*”: paths to transformation

**Figure 3:**
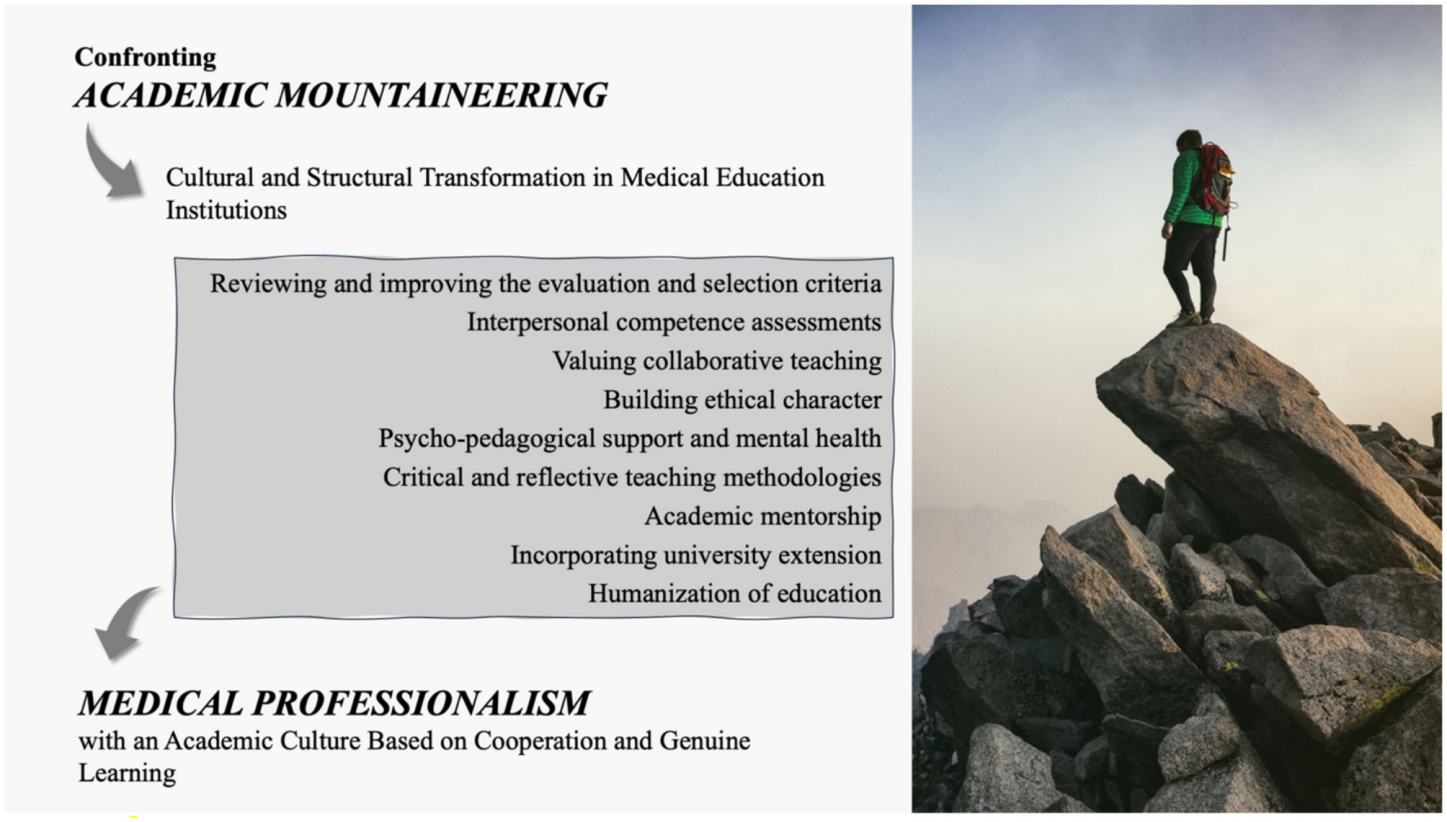
Ways of dealing with “*academic mountaineering*” and achieving success through medical professionalism through actions aimed at medical education.

Addressing “*academic mountaineering*” requires both a cultural and structural shift in medical education Figure 3. While this may be challenging, concrete changes can promote a more balanced and ethical approach to learning. One important step is to revise the evaluation and selection criteria at the undergraduate, residency, and postgraduate levels, focusing on genuine engagement and deep learning rather than just cognitive metrics. Assessments that prioritize clinical skills, ethics, and interpersonal abilities can redefine academic excellence and encourage more holistic training. Additionally, teaching methods that emphasize critical thinking, evidence-based practice, and clinical connections are essential. Open discussions on ethical dilemmas, coupled with mentoring, can help students place greater value on quality over quantity in their education.

The literature on institutional change highlights the importance of leadership, governance, and faculty development in driving sustainable reform. Without strategic investment in educator training and curricular autonomy, isolated initiatives are unlikely to succeed (40). Incorporating university extension into the curriculum, which integrates teaching, research, and community practice, strengthens the the humanization of training and promotes essential interpersonal and ethical skills. Additionally, enhancing mental health support programs with accessible services and self-care strategies is crucial. Educators and mentors trained to recognize signs of burnout can better support students in coping with academic pressure while maintaining their wellbeing. Transforming the educational environment requires a collective commitment to prioritizing humanistic, ethical training, restoring the values of medical professionalism, and fostering a culture of cooperation and authentic learning.

## 13 Conclusions

Medical training in Brazil faces significant challenges due to unchecked expansion of medical schools, intense competition, and an emphasis on *résume*-building over meaningful learning. “*Academic mountaineering*” promotes superficial knowledge, undermines professionalism, and negatively affects mental health. Its emergence can be seen not as a moral failure of students, but as a rational adaptation to institutional structures that reward appearances over substance. To restore the integrity of medical education, institutions must prioritize ethical values, humanistic learning, and comprehensive student support. Educational reform should be grounded in coherent pedagogical frameworks and supported by transparent governance and accountability mechanisms. Shifting the focus from quantity to quality will help produce well-rounded, competent, and compassionate doctors dedicated to the wellbeing of their patients and society.
